# Phenylephrine versus ephedrine on cerebral perfusion during carotid endarterectomy (PEPPER): study protocol for a randomized controlled trial

**DOI:** 10.1186/1745-6215-14-43

**Published:** 2013-02-14

**Authors:** Claire WA Pennekamp, Rogier V Immink, Wolfgang F Buhre, Frans L Moll, Gert Jan de Borst

**Affiliations:** 1 Department of Vascular Surgery (G04.129), University Medical Center Utrecht, P.O. Box 85500, Utrecht 3508, GA, The Netherlands

**Keywords:** Carotid endarterectomy, Cerebral oxygenation, Intraoperative hypotension, Phenylephrine, Ephedrine

## Abstract

**Background:**

Intraoperative arterial hypotension can lead to severe complications in patients undergoing carotid endarterectomy, in particular if cerebral auto-regulation is impaired. Short-acting agents, such as phenylephrine or ephedrine, commonly used to correct intra-operative hypotension, have different hemodynamic effects. Recently, it was reported that, in healthy anesthetized subjects with intact cerebral auto-regulation, frontal lobe cerebral tissue oxygenation declined after phenylephrine bolus administration, while it was preserved after ephedrine use (Br J Anaesth 107:209–217, 2011; Neurocrit Care 12:17–23, 2010). However, the effect of both agents in patients undergoing carotid endarterectomy is unknown. The aim of this study is to assess the effect of two routinely used vasopressors (phenylephrine and ephedrine) on the cerebral hemodynamics during carotid endarterectomy.

**Methods/design:**

Patients undergoing carotid endarterectomy will be prospectively included and randomized for correction of intraoperative hypotension with either phenylephrine (50 to 100 μg) or ephedrine (5 to 10 mg). If hypotension persists for more than five minutes after treatment, the patient will be classified as a non-responder and escape medication as preferred by the anesthesiologist will be administered. Changes in cerebral hemodynamics will be quantified by changes in transcranial Doppler-derived middle cerebral artery blood velocity and near infra-red spectroscopy-derived frontal lobe cerebral tissue oxygenation, when intra-operative hypotension is treated with phenylephrine or ephedrine in patients who undergo carotid endarterectomy with or without an adequate functioning cerebral auto-regulation.

To quantify whether the intra-operative cerebral auto-regulation is impaired or not, a decrease in breathing frequency from the normal 12 breaths per minute to 6 breaths per minute for an episode of three minutes will be performed.

**Discussion:**

Phenylephrine and ephedrine are two of the most commonly used short-acting agents to increase blood pressure in clinical anesthesiologic practice. Monitoring of middle cerebral artery blood velocity with transcranial Doppler and frontal lobe cerebral tissue oxygenation with near infra-red spectroscopy are part of the standard of care. Furthermore, there are no reports that the three-minute modification in breathing frequency described in the “intervention”-section is harmful. Therefore, the risks for participating patients are negligible and the burden minimal.

**Trial registration:**

Clinical trials.gov: NCT01451294

## Background

Carotid endarterectomy (CEA) is the recommended treatment to prevent future cardiovascular events in patients with a symptomatic high degree stenosis of the internal carotid artery (ICA). Besides these symptoms, stenosis of the ICA jeopardizes the cerebral perfusion and may affect cerebral auto-regulation (CA), indicating that cerebral perfusion becomes dependent on changes in blood pressure [1]. Therefore, to preserve cerebral perfusion during surgery and to prevent ‘watershed’ stroke, intraoperative hypotension needs to be avoided and the suggested blood pressure that should be maintained intraoperatively is an arterial pressure between baseline blood pressure measured on the nursing ward the day before surgery and 20% above this blood pressure [2].

To do so, different short-acting vasopressor agents can be used, such as phenylephrine or ephedrine [3]. If existing at all, preference for either of these agents is solely based on the discretion of the attending anesthesiologist. Furthermore, ephedrine and phenylephrine are widely accepted and, when heart rate is in the normal range, applied in cardiovascular surgery on the basis of individual preference. Both agents have a different mechanism of action. Phenylephrine (an α-agonist) increases blood pressure purely by vasoconstriction, whereas ephedrine (a combined α- and β-agonist) increases blood pressure by a combination of vasoconstriction and an increase in heart rate and a subsequent rise in cardiac output [4].

Besides the difference in systemic hemodynamics, the perfusion of the brain reacts differently. In healthy subjects, with intact CA, the frontal lobe cerebral tissue oxygenation (rSO_2_) decreases during phenylephrine administration while it is preserved with ephedrine use [4,5]. It is suggested that the increase in cardiac output observed during ephedrine use can explain this difference in rSO_2_[5]. It is unknown how in the situation of impaired CA, as often observed in patients undergoing a CEA, the rSO_2_ and vasomotor tone react after administration of phenylephrine or ephedrine. Thus, the optimal drug to maintain cerebral perfusion in CEA patients, with an impaired CA is unknown. If during the use of one of the two agents cerebral perfusion would be better maintained or even increased this would clearly influence the choice for the desired agent.

In our institution, we routinely monitor rSO_2_ using near infrared spectroscopy (NIRS) and middle cerebral artery blood velocity (*V*_MCA_) measured by transcranial Doppler (TCD) during CEA. To study the effect of both vasopressors on these parameters, we retrospectively analyzed the effect of phenylephrine and ephedrine induced changes in mean arterial pressure (MAP) on rSO_2_ and *V*_MCA_ in 11 CEA patients. We noticed that phenylephrine and ephedrine both increased MAP and *V*_MCA_ in patients undergoing carotid endarterectomy. However, an increase in MAP induced by phenylephrine has a lowering effect on the rSO_2_, while ephedrine had an increasing effect on the rSO_2_[6].

This pilot study indicated that the use of ephedrine should be preferred above the use of phenylephrine for correction of hypotension during CEA. However, the numbers of patients were small and the dose of agent applied not standardized. Therefore, a prospective study to analyze the effect of both ephedrine and phenylephrine on cerebral perfusion during CEA is needed to make a recommendation.

## Methods/ design

### Study objectives

Our primary objective is to study the influence of two routinely used drugs to increase MAP (phenylephrine and ephedrine) on cerebral oxygenation and perfusion, estimated by changes in NIRS derived rSO_2_ and TCD derived *V*_MCA_, in patients undergoing carotid endarterectomy.

Our secondary objective is to evaluate whether the influence of phenylephrine and ephedrine on cerebral perfusion and oxygenation is different between patients with and without an adequate functioning CA.

### Study design

#### Participants

This study is designed as a prospective randomized trial in a tertiary referral vascular center.

**Figure 1:**
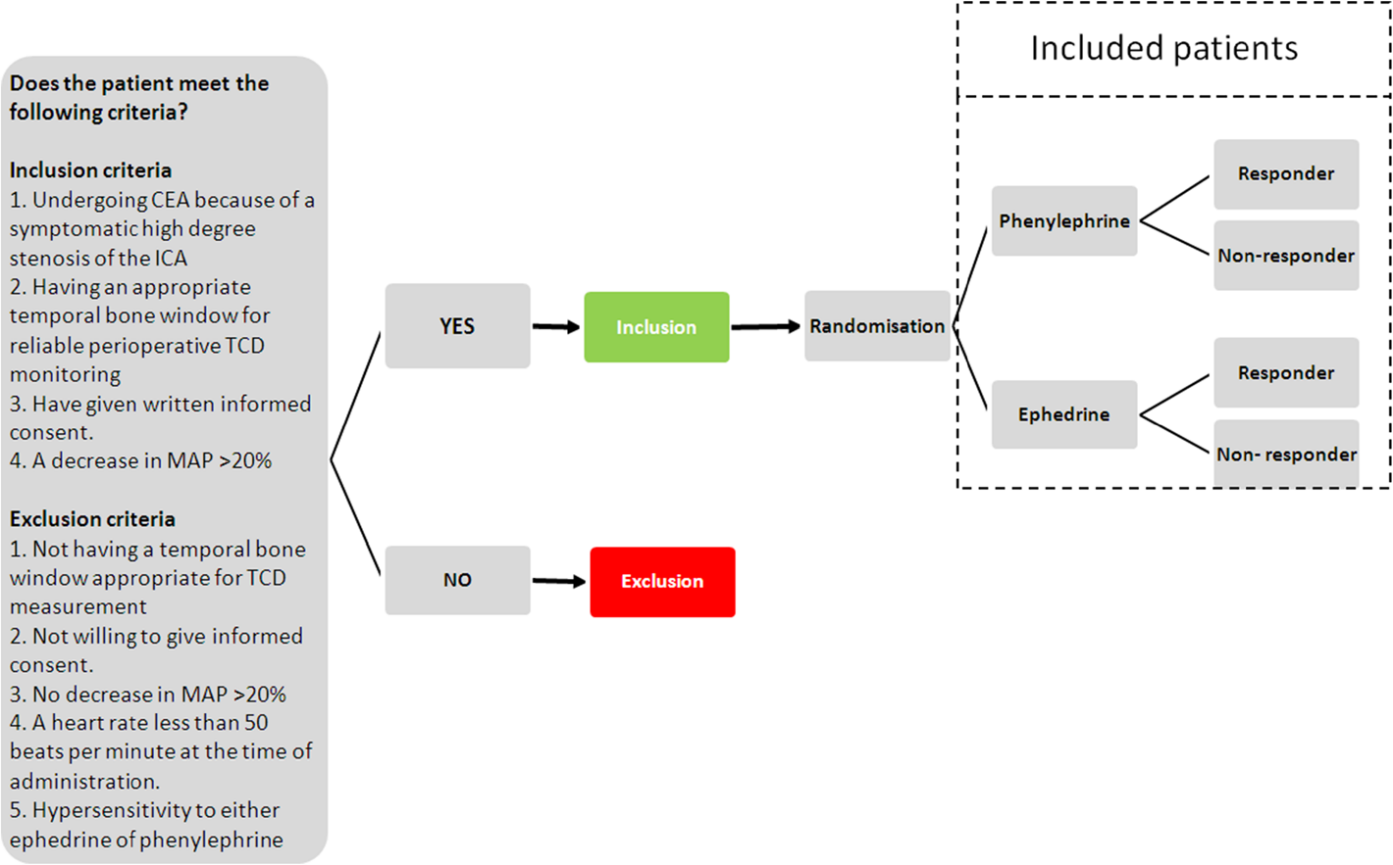
Study protocol flow chart.

The study population will include all patients indicated for CEA because of a symptomatic high degree stenosis of the ICA (discussed in a multidisciplinary meeting) in the University Medical Centre Utrecht (UMCU) who needs use of vasopressor to increase MAP during CEA [Figure 1].

To be included in the current study all patients must meet the following criteria:

1. Undergoing CEA because of a symptomatic high degree stenosis of the ICA in the University Medical Centre Utrecht.

2. Having an appropriate temporal bone window for reliable peri-operative TCD monitoring.

3. Having given written informed consent.

4. Having a decrease in MAP >20% during surgery.

Patients will be excluded if they meet one of the following criteria:

1. Not having a temporal bone window appropriate for TCD measurement.

2. Not willing to give informed consent.

3. No decrease in MAP >20%.

4. A heart rate less than 50 beats per minute at the time of administration.

5. Hypersensitivity to either ephedrine of phenylephrine.

#### Randomization and interventions

This study is designed as a prospective randomized trial in a tertiary referral vascular center. Currently, the preference of administering either phenylephrine or ephedrine for correction of intraoperative hypotension is mainly based on the physician’s preferences. For this trial randomization based on a computer-generated randomization list will be performed during CEA to counteract changes in MAP more than 20% under baseline with either

1) Phenylephrine (50 to 100 μg), or

2) Ephedrine (5 to 10 mg),

If hypotension persists five minutes after administration of the randomized medication, this patient will be classified as a non-responder and escape medication as preferred by the anesthesiologist will be administered.

Before surgery all patients will be informed about the trial procedure. Written informed consent will be obtained from all patients.

As part of the standard of care cerebral monitoring, including transcranial Doppler (TCD), Near Infrared Spectroscopy (NIRS) and electroencephalography (EEG) will be provided. To quantify whether the intra-operative cerebral auto-regulation is impaired or not, the breathing frequency will be decreased from the normal 12 breaths per minute to 6 breaths per minute for an episode of three minutes.

#### Study parameters

Our primary study parameters are the changes in rSO_2_ measured using NIRS and *V*_MCA_ measured using TCD. Our secondary parameter is quantification of cerebral auto-regulation.

#### Sample size calculation

To determine the sample size, we conducted a power analysis based on a retrospective pilot study. In this pilot study (performed in the UMCU), the response within each subject group was normally distributed. After phenylephrine administration a decrease in rSO_2_ of −1.5% (± 2) per 10 mmHg increase was seen, while after ephedrine use the rSO_2_ increased 1% (± 2) per 10 mmHg increase. We calculated that 14 patients in each group would be required to reject the null hypothesis that the population means of the experimental and control groups are equal with probability (power) 0.9. The Type I error probability associated with this test of this null hypothesis is 0.05. Adjusting the sample size to drop out of patients (because of bad signal quality, medical reasons), 20 patients per group will be sufficient.

In our vascular center, CEA is performed weekly. We assume two patients can be included weekly. Therefore, the estimated length of the study will be approximately six months.

### Medication

Ephedrine is a sympathomimetic amine acting directly on the alpha and β-receptors and indirectly by increasing the release of norepinephrine by the sympathetic nerve endings. As with any sympathomimetic agent, ephedrine stimulates the central nervous system, the cardiovascular system, the respiratory system and the sphincters of the digestive and urinary tract. After intravenous administration, ephedrine is completely biologically available. Intravenous injections are effective within a minute and for up to about 20 minutes. Small quantities of ephedrine are metabolized in the liver, but the majority of ephedrine is excreted unchanged in the urine. Elimination of ephedrine is increased (and hence the half-life is decreased) with decreasing pH of the urine.

Phenylephrine hydrochloride is a sympathomimetic agent with mainly direct effects on adrenergic receptors. It has predominantly α-adrenergic activity and is without significant stimulating effects on the central nervous system at usual doses. After injection it produces peripheral vasoconstriction and increased arterial pressure. It also causes a baro-reflex mediated bradycardia. Intravenous injections are effective within a minute and for up to about 20 minutes. Phenylephrine is metabolized in the liver by monoamine oxidase. The metabolites, their route and rate of excretion have not been identified.

For both ephedrine and phenylephrine the preparation and labeling will be conducted as in the pharmacy department of the UMC Utrecht. The investigational products are part of the standard of care and, therefore, received, stored and disposed at the pharmacy of the University Medical Center Utrecht.

### Intraoperative care

#### Anesthesia (no deviation of standard of care)

Following connection of the patient to the anesthesia monitor, an intra-arterial catheter in the radial artery is placed in each patient for arterial blood pressure measurement. Induction of anesthesia is achieved with sufentanil (0.3 to 0.7 mcg/kg) and propofol (0.5 to 2.0 mg/kg). Rocuronium (0.3 to 0.5 mg/kg) is used to facilitate tracheal intubation. Anesthesia is maintained using the volatile anesthetic sevoflurane, aiming at a Minimum Alveolar Concentration (MAC) value of 0.5 to 1. Anesthesia-related hypotension is treated with either phenylephrine or ephedrine administration (study medication). In case hypotension persists five minutes after administration of either ephedrine or phenylephrine, the patient will be classified as a non-responder and escape medication as preferred by the anesthesiologist will be administered. Outside the study period, the choice of treatment to correct hypotension will be based on the physician’s preferences. After heparinization, MAP is maintained between −10% and +10% from baseline, until carotid artery cross clamping is performed. At the end of the procedure, the sevoflurane supply will be stopped, and after return of spontaneous respiration, the trachea will be extubated and the patient will be transferred to the recovery ward.

#### Measurement of cerebral auto-regulation

To quantify whether the intra-operative CA is impaired or not, the breathing frequency is decreased from the normal 12 breaths per minute to 6 breaths per minute for an episode of three minutes. To maintain an unchanged minute alveolar ventilation of approximately six liters per minute, the tidal volume has to be almost doubled. The intra-thoracic pressure oscillations created with this way of ventilating will be transferred to the arterial blood pressure signal since a higher intra-thoracic pressure during mechanical insufflation of the lungs will prohibit blood from entering the thoracic cavity. Approximately two heartbeats after the start of the inspiration the amount of blood delivered to the right side of the heart decreases and also the amount of blood leaving the left side of the heart (that is, cardiac output) declines. In the expiration phase the opposite occurs and cardiac output increases. This variation in cardiac output results in a concomitant oscillation in the beat-to-beat registered blood pressure and this is again transferred to the beat-to-beat *V*_MCA_ and rSO_2_ signals.

Intact CA implies that the cerebral perfusion remains constant despite changes in blood pressure. Hypothetically, this means that the *V*_MCA_ would remain totally unaltered despite the 6 per minute blood pressure oscillations as elicited with the ventilator in anesthetized subjects. In reality, CA behaves like a high pass filter. This means that oscillations with a duration of 10 seconds in blood pressure will be present but dampened in the *V*_MCA_ signal. Furthermore, oscillations in *V*_MCA_ in this frequency area precede those in MAP by approximately two to three seconds. The better CA functions the more the amplitude of the oscillation in *V*_MCA_ will be dampened and the more the *V*_MCA_ signal precedes the blood pressure signal.

### Intraoperative monitoring (no deviation of standard of care)

#### Blood pressure monitoring

A radial artery catheter is placed in each patient for continuous blood pressure measurement.

Cardiac output monitoring:

Noninvasive arterial pressure (NAP) will be measured using the Nexfin monitor (BMEYE B.V., Amsterdam, The Netherlands), which uses an improved finger cuff technology with high sensitivity optical components and fully digital control systems. In addition, brachial arterial blood pressure is reconstructed from the measured finger arterial pressure using waveform filtering to approximate a brachial pressure wave, together with pressure level correction compensating for the finger to radial pressure difference. An appropriate size finger cuff will be applied to the mid-phalanx of the middle finger ipsilaterally to the invasive blood pressure catheter. The “heart reference system”, which measures and corrects the hydrostatic difference between the finger and the heart, will be set at the arterial pressure transducer level.

#### Near-infrared spectroscopy

Bilateral rSO_2_ measurements will be performed using Invos Cerebral Oximeter (Somanetics Corporation, Troy, MI, USA), which allows continuous monitoring of cerebral oxygenation, by penetrating the scalp and brain tissue whereby the skin, scull and other tissues are relatively transparent to near-infrared wavelengths of light. Changes in concentrations of oxygenated and deoxygenated hemoglobin are measured by a modified Beer-Lambert method and because of the different wavelengths of the oxygenated and deoxygenated hemoglobin, these can be distinguished and the ratio of oxygenated hemoglobin to total tissue hemoglobin (TOI) can be defined. By using two detectors the light reflected and transmitted by the superficial extracranial tissues is subtracted.

#### TCD measurement

For all TCD registrations a pulsed Doppler transducer (DWL Multidop X4, Sipplingen, Germany) gated at a focal depth of 45 to 60 mm, will be placed over the temporal bone to insonate the main stem of the ipsilateral middle cerebral artery and the contralateral anterior cerebral artery.

After tracheal intubation, TCD is applied. The TCD probes are fitted in a light metal frame, which is firmly fixed to the head with two earpieces and an adjustable nose saddle. The *V*_MCA_, will be measured continuously.

#### EEG measurement

For measuring the cerebral function state and detecting signs of cerebral ischemia, used as an indication for selective shunting during CEA, electroencephalography (EEG; Micromed Inc., Treviso, Italy) is used. Prior to starting anesthesia, EEG electrodes will be applied to the patient’s skull and the EEG will be continuously registered during surgery.

### Carotid endarterectomy (no deviation of standard of care)

A vascular surgeon or vascular trainee under supervision will perform carotid surgery in a standardized way. Patients will receive 5,000 IU heparin three minutes before cross-clamping the carotid artery. The decision for a venous, prosthetic or bovine patch will be made by preference of the surgeon. An intra-luminal shunt will selectively be used based on TCD and/ or EEG criteria as described in the literature [7].

### Recruitment and consent

The principal investigator, who will inform individual participants about the study, will do patient recruitment. Informed consent will be obtained as soon as possible, but must be acquired before surgery. The patient information letter and informed consent form are attached as a separate document.

### Withdrawal of individual subjects

Subjects can leave the study at any time for any reason if they wish to do so without any consequences. The investigator can decide to withdraw a subject from the study for urgent medical reasons. Any patient with incomplete data registration can be withdrawn and replaced by a consecutive patient.

### Follow-up of subjects

Patients will receive no follow-up other than regular follow-up procedures following carotid endarterectomy (outpatient clinic visit combined with duplex ultrasound of the operated carotid bifurcation).

### Adverse and serious adverse events

Adverse events, serious adverse events and suspected, unexpected, serious adverse reactions will be recorded and reported according to the requirements of the accredited medical ethical committee.

### Statistical analysis

The MAP, TCD and NIRS measurements will be presented as continuous variables for both of the randomized groups.

To examine the effect of either ephedrine or phenylephrine on rSO_2_ and *V*_MCA_ an intention-to-treat analysis will be used. For this analysis all patients that received either ephedrine or phenylephrine will be included (both responders and non-responders). Subsequently, a sub analysis will be performed to compare the changes in rSO_2_ and *V*_MCA_ per 1 mmHg increase between patients that received either ephedrine or phenylephrine. For this sub analysis only the responders will be included.

To compare the relationship between the phenylephrine and ephedrine group in an rSO_2_ and *V*_MCA,_ the Student’s *t*-test will be used. The relationship will be calculated at two different time points:

• two minutes after either phenylephrine or ephedrine administration (t_2_) and

• at the moment the maximum increase in blood pressure is reached (t_max_).

To determine whether the influence of phenylephrine and ephedrine on cerebral perfusion and oxygenation is different between patients with and without an adequate functioning cerebral auto-regulation, another subanalysis within both groups will be performed using a Student’s *t*-test.

The statistical analysis will be performed using the Statistical Package for Social Sciences version 20.0 (SPSS Inc, Chicago, Il, USA). A confidence level of less than 5% (0.05) is considered significant.

## Discussion

Since both phenylephrine and ephedrine have been routinely used for years and all measurements are part of the standard of care, no side effects are expected. Furthermore, there are no reports that the three-minute modification in breathing frequency described in the “intervention”-section is harmful. Varying breathing frequencies while maintaining minute ventilation results in larger tidal volumes and, therefore, an unchanged oxygen uptake and carbon dioxide remove, which can be measured using a pulse oximeter. Larger tidal volumes, however, result in higher airway pressures during low frequency ventilation. Inspiratory and expiratory airway pressures in healthy lungs during 6 mL·kg^-1^ with a normal ventilatory frequency of 12 min^-1^ are approximately 15 and 5 mmHg, respectively. During a ventilatory frequency of 6 min^-1^ inspiratory and expiratory airway pressures will rise to approximately 25 and 5 mmHg. This is comparable to airway pressures observed during laparoscopic surgery when the abdomen is inflated with carbon dioxide or during surgery with the body in 20° Trendelenburg position.

Therefore, the risks for participating patients are negligible and the burden minimal.

To our best knowledge this trial represents the first attempt to evaluate the differential effects of phenylephrine and ephedrine in correction of intraoperative hypotension during CEA. If either of these drugs is shown to be superior with respect to the cerebral hemodynamics, results from our study will provide clinical trial evidence for the management of blood pressure during CEA.

### Trial status

The Medical Ethics Committee (METC) of the University Medical Center Utrecht has approved this study protocol. The trial has already been started in October 2012; the estimated length of the study will be six months.

## Abbreviations

CA: Cerebral auto-regulation; CEA: Carotid endarterectomy; EEG: Electroencephalography; ICA: Internal carotid artery; MAC: Minimum Alveolar Concentration; MAP: Mean arterial pressure; METC: Medical Ethics Committee; NAP: Noninvasive arterial pressure; NIRS: Near infrared spectroscopy; rSO_2_: The frontal lobe cerebral tissue oxygenation; TCD: Transcranial Doppler; TOI: The ratio of oxygenated hemoglobin to total tissue hemoglobin; UMCU: University Medical Centre Utrecht; *V*_MCA_: Middle cerebral artery blood velocity.

## Competing interests

The authors declare they have no competing interests in relation to this manuscript.

## Authors’ contributions

CP participated in the design of the study and drafted the manuscript. RV and GB participated in the design of the study and helped to draft the manuscript. WB and FM participated in the design of the study and critically revised the manuscript. All authors read and approved the final manuscript.
